# Buffer mobility and the regulation of neuronal calcium domains

**DOI:** 10.3389/fncel.2015.00048

**Published:** 2015-02-20

**Authors:** Elizabeth A. Matthews, Dirk Dietrich

**Affiliations:** Experimental Neurophysiology, Department of Neurosurgery, University Clinic BonnBonn, Germany

**Keywords:** calcium buffer, mobile, immobile, calcium domains, diffusion coefficient

## Abstract

The diffusion of calcium inside neurons is determined in part by the intracellular calcium binding species that rapidly bind to free calcium ions upon entry. It has long been known that some portion of a neuron’s intracellular calcium binding capacity must be fixed or poorly mobile, as calcium diffusion is strongly slowed in the intracellular environment relative to diffusion in cytosolic extract. The working assumption was that these immobile calcium binding sites are provided by structural proteins bound to the cytoskeleton or intracellular membranes and may thereby be relatively similar in composition and capacity across different cell types. However, recent evidence suggests that the immobile buffering capacity can vary greatly between cell types and that some mobile calcium binding proteins may alter their mobility upon binding calcium, thus blurring the line between mobile and immobile. The ways in which immobile buffering capacity might be relevant to different calcium domains within neurons has been explored primarily through modeling. In certain regimes, the presence of immobile buffers and the interaction between mobile and immobile buffers have been shown to result in complex spatiotemporal patterns of free calcium. In total, these experimental and modeling findings call for a more nuanced consideration of the local intracellular calcium microenvironment. In this review we focus on the different amounts, affinities, and mobilities of immobile calcium binding species; propose a new conceptual category of physically diffusible but functionally immobile buffers; and discuss how these buffers might interact with mobile calcium binding partners to generate characteristic calcium domains.

Calcium is a broadly active signaling molecule in neurons and can initiate a diverse set of actions, from neurotransmitter release, to induction of synaptic plasticity, to gene transcription. But intracellular calcium is not indiscriminately active, indicating that systems for controlling and directing the signaling cascade must exist. This regulation results from the combined effects of localized calcium entry, binding by mobile and immobile endogenous buffering species, distribution within intracellular compartments, and finally removal from the intracellular space. In combination, these regulatory actions produce different spatial and temporal domains of calcium: microdomains around single channels, calcium signals limited to spines or small dendritic segments, and global calcium elevations involving the entire cell (Augustine et al., 2003). Calcium entry, binding, diffusion, and removal have been topics of intense focus in order to better predict the types of calcium domains that could arise during physiological neuronal activity and to better understand the functional consequences of each calcium domain on information transfer and the pattern of pre- and post-synaptic activity. In the period of time after calcium has entered a cell, but before it has been removed or sequestered into internal stores, the distribution and availability of calcium is dictated by the presence of endogenous buffering molecules.

## CONSIDERING BUFFER MOBILITY

Initially, endogenous calcium buffers were assumed to be “unsaturable, infinitely fast, and immobile” (Sala and Hernandez Cruz, 1990). However, the development of fast calcium dyes that allowed for direct measures of the intracellular free calcium concentration in real time made it clear that there must be fast buffers present in cells that could be washed out relatively rapidly by the patch pipette (Thayer and Miller, 1990; Zhou and Neher, 1993; Mueller et al., 2005), and were thus mobile. The impact of mobile buffers on the time course and spatial range of calcium signals was first shown by modeling (Sala and Hernandez Cruz, 1990), and later experimentally. Since then, the molecular identities of many of these mobile binding partners have been uncovered, and their binding properties have been intensely studied. The properties and functional role of mobile calcium binding proteins have been well reviewed (Baimbridge et al., 1992; Schwaller, 2010), and so this review will instead focus attention on the somewhat neglected topic of immobile calcium buffers in neurons. Immobile buffers, although their molecular identities remain unknown, deserve separate consideration because of their unique role in shaping the spatial domain of intracellular free calcium. Immobile buffers alone greatly slow the spatial spread of calcium, while at the same time prolonging the temporal duration of the signal. In the presence of mobile buffers, immobile buffers increase the complexity of the spatio-temporal signaling repertoires available to the neuron, based on the relative affinities, kinetics, and concentrations of the different buffers.

At the outset, we should define “immobile.” Simplistically, the physical definition is a buffer that has a diffusion coefficient of 0 μm^2^/s, while a mobile buffer has a diffusion coefficient >0 μm^2^/s. Of more interest though, is the functional mobility, i.e., how does the buffer affect the amount and distribution of free calcium. Under certain physiological conditions, even mobile buffers can function like an immobile buffer by slowing and restricting the increase and spread of free calcium concentration. Even a highly mobile buffer retards calcium diffusion relative to the diffusion of unbound calcium (∼220 μm^2^/s for free calcium in cytosol (Allbritton et al., 1992), implying that mobile buffers will also slow calcium diffusion and can act analogously to immobile buffers. In contrast, small exogenously applied calcium chelators such as BAPTA, Oregon Green-BAPTA, or EGTA that are commonly used in experiments will virtually always function as mobile buffers, as their estimated diffusion coefficients are nearly the same as for free calcium (Naraghi and Neher, 1997). So in what context is a mobile buffer functionally immobile? Using Equation 1, presented later in this paper, we have calculated buffer diffusion coefficient threshold values to predict, whether the effect of a generic diffusible buffer on free calcium will be that of a functionally mobile or immobile buffer within three spatial domains representing microdomains around a channel, in a spine-like compartment, or in a dendritic segment. Buffer capacity is quantified by the unit-less measure κ (see following paragraphs). With a background of high immobile buffer capacity (κ_immobile_ = 150), the mobile buffer must have a diffusion coefficient greater than 2 μm^2^/s to increase the apparent diffusion coefficient of calcium (D_app_) by ∼10%. However, in the context of low immobile buffering (κ_immobile_ = 15), even a mobile protein with a diffusion coefficient of ∼14 μm^2^/s would act as a functionally immobile buffer, slowing the apparent diffusion of calcium. Many well-known calcium binding proteins (parvalbumin, calbindin, etc) are mobile, with diffusion coefficients that have been measured in cells and *in vitro*. The diffusion of mobile proteins varies between sub-cellular compartments (Schmidt et al., 2003, 2007), and in the case of calretinin, following neuronal activity (Arendt et al., 2013); these measured diffusion coefficients cover a large range [calretinin: 2.2 μm^2^/s (Arendt et al., 2013), calbindin: 20 μm^2^/s (Schmidt et al., 2005), parvalbumin: 43 μm^2^/s (Schmidt et al., 2007)]. For the mobile proteins at the slower end of the range, whether they function as mobile or immobile at the microdomain level depends entirely on the background of physically immobile buffer that is concurrently available.

## THEORETICAL DEFINITION AND EXPERIMENTAL QUANTIFICATION OF BUFFERING

Buffering capacity (κ) describes how much calcium will be bound and how much will remain free following an increase in free calcium inside the neuron. It is typically quantified at resting calcium concentration of the cell (**Box 1**). A high buffering capacity means that very little calcium will remain free following an action potential or other calcium-generating event. Numerous studies have examined the total endogenous buffer capacity present in various cell types, although because of the different experimental approaches, comparisons between the reported binding capacities are complicated (see **Table 1**). Further confusing the issue, the relative contributions of the mobile and immobile buffering fractions have not always been directly or independently measured in these different studies. In some cases, the authors disregarded the question of buffer mobility entirely.

**Table 1:** Endogenous buffering capacity reported in different neuron types.

Cell type	Endogenous κ	Evidence of mobility/immobility	*K*_d_ (μM)	Reference
**Hippocampus**
CA1 pyramidal spine	14–22	Added buffer (no duration mentioned); dye loading (no check of γ)		Sabatini et al. (2002)
CA1 pyramidal dendrite	28–32	Added buffer (no duration mentioned); dye loading (no check of γ)		Sabatini et al. (2002)
CA1 pyramidal dendrite	27	Dye loading (no check of γ)		Rozsa et al. (2004)
CA1 pyramidal dendrite (adult)	41–101	Dye loading (no check of γ)		Maravall et al. (2000)
CA1 pyramidal dendrite (juvenile)	24–68	Dye loading (no check of γ)		Maravall et al. (2000)
Cultured hippocampal neuron (excitatory)	57–60	Dye loading (A*τ, no check of γ; re-patch after bolus loading		Lee et al. (2000)
CA1 pyramidal dendrite	101	Dye loading (A*τ, no check of γ)		Liao and Lien (2009)
CA1 pyramidal dendrite	168–207	Added buffer (10 min duration); dye loading (A*τ, no check of γ; bolus load (1 min duration)		Helmchen et al. (1996)
DG granule cell axon	17–25	Added buffer (no duration mentioned)	0.5	Jackson and Redman (2003)
DG granule cell dendrite (adult)	150–300	Added buffer (40 min duration); re-patch after bolus loading		Stocca et al. (2008)
DG granule cell dendrite (juvenile)	50–100	Added buffer (40 min duration); re-patch after bolus loading		Stocca et al. (2008)
DG granule cell soma	200	Quantitative immunohistochemistry; replacement with purified calbindin		Mueller et al. (2005)
DG granule cell dendrite	90–124	Added buffer (40 min duration); Cb knockout		Matthews et al. (2013)
DG granule cell dendrite	150–300	Added buffer (8 min duration)		Matthews et al. (2013)
Hippocampal OLM interneuron	15–33	Dye loading (A*τ, no check of γ)		Liao and Lien (2009)
Hippocampal OLM interneuron	28–31	Added buffer (comparison of 8 and 40 min duration)		Matthews et al. (2013)
Hippocampal CCK+ interneuron	65–82	Added buffer (no duration mentioned)		Evstratova et al. (2011)
St. Radiatum interneuron	71	Dye loading (no check of γ)		Rozsa et al. (2004)
Cultured hippocampal interneuron	130–150	Dye loading (A*τ, no check of γ; re-patch after bolus loading		Lee et al. (2000)
Schaffer collateral interneuron	171–187	Added buffer (no duration mentioned)		Evstratova et al. (2011)
DG basket cell	202–214	Added buffer (20–30 min duration); dye loading (no check of γ)		Aponte et al. (2008)
**Cortex**
Cortical pyramidal neuron (spine)	19	Added buffer (20–30 min duration)		Cornelisse et al. (2007)
Cortical pyramidal neuron dendrite	62	Added buffer (20–30 min duration)		Cornelisse et al. (2007)
Layer 5 pyramidal neuron	105–135	Added buffer (10 min duration); dye loading (A*τ, no check of γ; bolus load (1 min duration)		Helmchen et al. (1996)
Layer 2/3 pyramidal neuron	185	Added buffer (no duration mentioned); dye loading (no check of γ)		Kaiser et al. (2001)
Cortical bitufted interneuron	285	Added buffer (no duration mentioned); dye loading (no check of γ)		Kaiser et al. (2001)
**Cerebellum**
Purkinje neuron	2000	Added buffer (no duration mentioned); dye loading (no check of γ)		Fierro and Llano (1996)
Cultured Purkinje neuron	1200–2000	Plotting free Ca^2+^ vs. total Ca^2+^ (no check of γ)		Maeda et al. (1999)
**Other regions/cell types**
Calyx of held	26–71	Dye loading (no check of γ)		Helmchen et al. (1997)
Substantia nigra DA neuron (juvenile)	96–117	Dye loading (A*τ, no check of γ)	0.2–0.3	Foehring et al. (2009)
Substantia nigra DA neuron (adult)	176	Dye loading (A*τ, no check of γ)	0.2–0.3	Foehring et al. (2009)
Retinal bipolar cell	720	Plotting free Ca^2+^ vs. total Ca^2+^ (no check of γ)	2	Burrone et al. (2002)
Retinal rod cell	25	Added buffer (no duration mentioned)		Van Hook and Thoreson (2014)
Retinal cone cell	50	Added buffer (no duration mentioned)		Van Hook and Thoreson (2014)
**Bovine adrenal cells**
Chromaffin cell	30–55	Plotting free Ca^2+^ vs. total Ca^2+^ (no check of γ)		Naraghi et al. (1998)
Chromaffin cell	40	Plotting free Ca^2+^ vs. total Ca^2+^ (no check of γ)	100	Xu et al. (1997)
Chromaffin cell	40	Re-patch after bolus loading; perforated patch	>2	Zhou and Neher (1993)
Chromaffin cell	75	Dye loading and unloading (no check of γ)	>1	Neher and Augustine (1992)
**Mollusc neurons**
Sea slug neuron	18.5	Plotting free Ca^2+^ vs. total Ca^2+^ (no check of γ)		Ahmed and Connor (1988)
Aplysia axon	20–60	Measured *D_app_* of Ca^2+^ with different dye concentrations; sharp microelectrode		Gabso et al. (1997)
Sea slug axoplasm	50–100	Plotting free Ca^2+^ vs. total Ca^2+^ (no check of γ)		al-Baldawi and Abercrombie (1995a)

**Box 1**

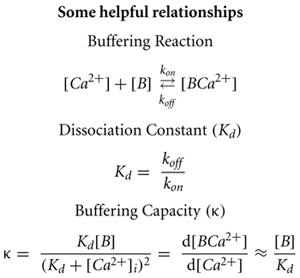

The “added buffer” method is typically used to measure the endogenous buffer capacity. This method involves the addition of exogenous calcium buffer (dye or chelator) via a patch pipette. The endogenous κ value is then extrapolated from the linear relationship found by plotting amplitude or decay of a calcium transient against the added buffer. The experiment can be done in either a population of cells or in a single cell. In population experiments, different cells are loaded with different, known amounts of exogenous buffer. If the measures of endogenous buffering are repeated at fixed time points during the recording, then changes in endogenous buffering (due to washout), run-down or run-up of extrusion, and changes in calcium entry are easily identified by comparing the fitted data from early in the experiment to later time points. The second approach is to load dye into a single cell and use the increasing exogenous κ during the initial loading period to extract the endogenous buffer capacity. Neither of these methods can inherently differentiate between the contributions of mobile and immobile buffers to the total endogenous κ. If the recording durations, the distance of the imaging location from the patch pipette, and the expression of mobile calcium binding proteins are reported, an educated guess can be made as to the extent of washout of the mobile fraction (Pusch and Neher, 1988; Scott and Rusakov, 2006). In the absence of such specific information or an explicit check of the mobility of the endogenous buffering species, the total endogenous κ reported by the added buffer method sets an upper limit for the amount of immobile κ present – either the reported buffer capacity is entirely immobile, or the reported value represents a mixture of mobile and immobile components, and the immobile buffering capacity is less than the measured value.

Most studies using the dye loading method report loading times between 3 and 10 min for proximal dendrites to achieve a plateau fluorescence; even with these short loading times, mobile buffers may be lost to the recording pipette, especially from proximal sites (Mueller et al., 2005). Some of these studies have used the product of the transient amplitude and decay time constant (A^∗^τ), which has been assumed to be constant throughout the experiment (Helmchen et al., 1996; Lee et al., 2000), to ensure that there are not alterations in calcium entry or extrusion (γ) during the dye loading period and that the decay time constant or the amplitude truthfully reflect the total buffer concentration. However, if both entry and extrusion change in tandem as has been observed (Matthews et al., 2013; see Discussion), the product A^∗^τ may remain constant but neither τ nor A would truthfully reflect the total buffer concentration and may mask loss of endogenous buffer. The population approach, by virtue of allowing measurements to be taken across the cell population at identical experimental time points, ensures that calcium extrusion and entry are the same for all “added” calcium dye values, and thus is an uncontaminated measure of the endogenous buffering capacity, and thus τ and A can be used to assess the total endogenous buffer capacity. Note that, long recording times do not guarantee that only immobile buffering species are left; it can take over 45 min to fill the fine distal dendrites or axons with dye, and a large protein is unlikely to be washed out more rapidly than a small dye molecule can be loaded (Scott and Rusakov, 2006).

In hippocampal pyramidal neurons, values for the total endogenous κ range from 15 to 20 (Sabatini et al., 2002; Rozsa et al., 2004) to 150–200 (Helmchen et al., 1996). For cortical and hippocampal interneurons the reported κ values range from 15 to 30 (Liao and Lien, 2009; Matthews et al., 2013) to 285 (Kaiser et al., 2001). The highest reported value is for Purkinje neurons, with a measured calcium buffering capacity of ∼2000 (Fierro and Llano, 1996; Maeda et al., 1999). It is therefore clear that κ varies strongly between cell types and even between different sub-compartments (spines, axon boutons) within the same cell (Sabatini et al., 2002; Jackson and Redman, 2003). All of these factors make it difficult to ascertain the source of the large variances between cell types – are these differences due to different measurement techniques, or the whole or partial removal of mobile buffering species, or are they integral to the spatio-temporal calcium profiles of the different neurons? Clearly, the expression of calcium binding proteins will vary from cell type to cell type, and may even vary at different developmental states of the neurons, as calbindin does for dentate granule cells (Yoon et al., 2000); it has not been as obvious whether reported differences in the immobile buffering components are similarly cell type specific.

The most reliable measures of immobile buffering capacity come from studies that have explicitly separated the mobile and immobile fraction, by either long duration recordings that monitor for a change in buffering capacity over time, or use of perforated patch recordings to prevent washout followed by whole cell recording. Applying this strict criteria to the list in **Table 1** results in an estimate of endogenous immobile buffering capacity of 90–124 in dentate gyrus granule cells (Matthews et al., 2013); 28–31 in hippocampal OLM interneurons (Matthews et al., 2013); and 40–75 in bovine chromaffin cells (Zhou and Neher, 1993) Other studies have made concerted efforts to detect a change in buffering capacity over the course of the recording by comparing the endogenous buffering after very short recordings to measures made after the longer dye-loading process. Including these measurements suggests that the immobile fraction of the total buffer capacity for CA1 pyramidal neurons is 168–207 (Helmchen et al., 1996); 105–135 in Layer V pyramidal neurons (Helmchen et al., 1996); and 285–288 in cortical bi-tufted interneurons (Kaiser et al., 2001). These measures provide evidence that endogenous immobile buffering capacity is not a general property of cells, but is tailored to the cell type and must play a role in setting different calcium signaling regimes from cell type to cell type.

## ADDITIONAL PROPERTIES OF THE IMMOBILE BUFFER

The effect of any buffer on the calcium distribution does not solely depend on κ. The affinity, concentration, and on-rate of the buffer are also important, especially if several buffering species compete for the available free calcium (Markram et al., 1998; Helmchen and Tank, 2005). For instance, a buffer with a slow on-rate will have difficulty impacting the local calcium concentration, even if it is present in high concentration, because the calcium cloud can disperse through diffusion before the buffer has time to bind many molecules (Eggermann and Jonas, 2012). In addition to ascertaining the immobile endogenous buffering capacity, it is also important to consider the probable affinity, concentration, and speed of the calcium binding species that contribute to the immobile fraction. Unfortunately, the literature on affinity, concentration and on-rate of the endogenous immobile buffer is sparse. The most comprehensive studies were done by the group of Neher using bovine adrenal chromaffin cells. They reported an immobile buffering capacity of ∼30 and a dissociation constant (*K*_d_) greater than 2 μM in these cells (Neher and Augustine, 1992; Zhou and Neher, 1993). In a later study, they refined the *K*_d_ measurement and report a dissociation constant of 100 μM and a concentration of 4 mM for the immobile buffer (Xu et al., 1997). This is in good agreement with other indicators that the immobile buffer would be low affinity based on the equivalent saturation of BAPTA (Tillotson and Gorman, 1980; Lumpkin and Hudspeth, 1998) and the constancy of the buffering capacity with large elevations in calcium (Thayer and Miller, 1990; Allbritton et al., 1992; al-Baldawi and Abercrombie, 1995b). If the resting calcium level is much smaller than the K_d_ for a given buffer, then κ can be approximated as the concentration of the buffer divided by the K_d_ (**Box 1**). The resting calcium concentration in neurons is reported to be ∼20–100 nM (Schwaller, 2010), which is much less than even the smallest estimated value for the K_d_ of the endogenous immobile buffer. We can therefore estimate a range for the concentration of this buffer as between 90 μM (κ = 30, *K*_d_ = 3 μM) and 12 mM (κ = 120, *K*_d_ = 100 μM). The kinetics of calcium binding by the immobile buffer are also unclear; the on-rate has only been measured directly in bovine chromaffin cells and was reported to be 1.07 × 10^8^ M^-1^s^-1^ (Xu et al., 1997). The experimental data from other neurons suggests that the on-rate of the endogenous buffers must be fast enough to compete with typical calcium indicators. EGTA has a relatively slow on-rate: 2.5 × 10^6^ M^-1^s^-1^, while BAPTA and Fura have faster on-rates in the range of 5 × 10^8^ M^-1^s^-1^ (Naraghi et al., 1998). A conservative estimate of the on-rate for the endogenous immobile buffer would be in the range of 10^7^–10^8^ M^-1^s^-1^.

## POTENTIAL CANDIDATES CONTRIBUTING AN IMMOBILE BUFFERING CAPACITY

What molecular species might contribute this immobile buffering? Because of indications that the buffering in the middle of cells was lower than near the cell membrane (Tillotson and Gorman, 1983; Klingauf and Neher, 1997; Naraghi et al., 1998), it has been suggested that the immobile buffer might be a combination of negatively charged phospholipid groups on the intracellular face of the membrane (McLaughlin et al., 1981), in particular, phosphatidylserine, which is enriched in neurons (Kim et al., 2014). The affinity of these groups is indeed quite low: the estimated K_d_ of phospholipids for calcium is 10–80 mM (McDaniel and McLaughlin, 1985). However, there is no evidence that the membranes of different cell types would contain different enough amounts of phospholipids to create such a marked variation in buffering capacity as has been observed, and the concentration would have to be quite high for such a low affinity buffer to achieve a buffering capacity in the observed range (1500– 10,000 mM). Another possibility is that the immobile calcium buffering capacity is contributed by a heterogeneous mix of proteins which sense or bind calcium such as the calcium sensing domains of calcium dependent ion channels, cytoskeletal molecules and transport motors, or membrane-associated calcium binding kinases (Smith et al., 1996; Schwaller, 2010). This suggestion would fit the observation that immobile buffering capacity varies markedly between cell types, and can even vary between different regions in the same cell (spines vs. dendrites). To date, direct experimental evidence to either support or contradict the idea that the immobile buffer is composed of an assortment of phospholipids and calcium sensing membrane proteins is lacking.

Mitochondria are sometimes suggested as a potential candidate for the immobile calcium binding species. It is true that the majority of mitochondria are immobile (50–85%), and the fraction of mobile mitochondria decreases to approximately 5% when internal calcium is elevated above 400 nM (Wang and Schwarz, 2009). It would therefore be expected that all mitochondria in the vicinity of calcium channels or calcium permeable receptors will become immobile during neuronal firing. In addition to their role in energy production, mitochondria remove calcium from the intracellular space via their uniporters (Gunter and Pfeiffer, 1990). The uniporter has a low affinity for calcium, estimated at ∼10 μM (Patterson et al., 2007; Santo-Domingo and Demaurex, 2010). The rate of removal from the cytosol depends on the intracellular free calcium concentration, but the maximal rate has been estimated in the range of 4–225 μM/s when intracellular calcium is in the micromolar range (Babcock et al., 1997; Montero et al., 2000). When intracellular free calcium is strongly elevated, mitochondria may account for the majority of calcium binding and removal (Herrington et al., 1996). However, at calcium levels more commonly occurring in neurons, calcium uptake from the cytosol by mitochondria is considerably slower (Kann and Kovacs, 2007), and would not strongly compete with most endogenous buffers. Calcium is also extruded from mitochondria, primarily via a Na^+^/Ca^2+^ exchanger in the inner mitochondrial membrane (White and Reynolds, 1995; Kann and Kovacs, 2007), which can prolong the cytosolic elevation of calcium (Herrington et al., 1996; Babcock et al., 1997). Although mitochondria bind and release calcium in a calcium-dependent fashion, the mechanisms responsible for each action are independent of each other; the uniporter responds to the cytosolic calcium concentration, while the Na^+^/Ca^2+^ exchange responds to mitochondrial calcium concentration and cytosolic Na^+^ concentration. Because the calcium uptake and release are realized through distinct protein actors, and are coupled via intra-mitochondrial calcium, the application of classical biochemical treatments of buffers is not warranted. Mitochondria do play a role in supporting and shaping neuronal calcium signals, especially under conditions of strongly elevated cytosolic free calcium, but are not fast enough to remove a substantial portion of calcium within a physiologically relevant time window of a couple hundred milliseconds and thus are not a good candidate for the immobile buffer shaping calcium signals in the context discussed here.

## MODELING THE ROLE OF IMMOBILE BUFFERS IN THREE SCENARIOS

Thus far, the accumulated experimental evidence suggests that the endogenous immobile buffer varies by cell type, but in general has a buffering capacity in the range of ∼30–120, a low affinity with a dissociation constant in the range of 3–100 μM, and an on-rate similar to BAPTA. In the next section, we present an estimate of the impact of functionally immobile buffers on free calcium, exclusively. Whether and how the mobility or immobility of a buffer is important for its effect on free calcium depends on the spatial domain under consideration. We differentiate three scenarios: calcium signals spreading along dendrites, calcium signals with no relevant spatial concentration gradients, and submicroscopic calcium signals (microdomains). For our calculations, we assume that every cell has at least a minimal immobile buffering capacity of 15. This is justified by our previous work (Matthews et al., 2013) and also by the fact that none of the other previous studies found a lower total κ value in any type of neuron (**Table 1**). Only recently it was highlighted that the immobile buffer content may be significantly higher in certain types of neurons, which strongly affects the competition for calcium between mobile and immobile buffers (Matthews et al., 2013). We therefore also consider a condition with high immobile buffer capacity of 150. The other parameter values were: *k*_on_ = 1 × 10^8^ M^-1^s^-1^ and *K*_d_ = 5 μM. The immobile buffering capacity was assumed to be evenly distributed in the space. For simplicity, when mobile buffers were simulated with immobile buffers, the *k*_on_ and *K*_d_ were the same for both buffers.

In our first scenario, calcium diffusing along a dendrite, the spatial domains are sufficiently large, so that diffusional equilibrium takes longer than local chemical equilibrium between calcium and its binding partners. Such domains typically arise from a local calcium source in a dendrite with ensuing spread of the calcium ions along the dendrite. The diffusion of calcium within three dimensional space in the presence of multiple buffers of mixed mobility is a complex phenomenon, described by a set of partial differential equations, which can be linearized to describe individual interactions of calcium ions with buffers at the nano- and micro-domain level (Zador and Koch, 1994; Naraghi and Neher, 1997). An additional simplification assumes that interactions between calcium and buffers are instantaneous (Rapid Buffer Approximation) and that the spatio-temporal localization of calcium depends only on the diffusion coefficients and affinities of the various buffers (Wagner and Keizer, 1994; Smith et al., 1996; Neher, 1998). This yields a very useful analytical expression, which describes calcium diffusion in the presence of multiple buffers using a new, smaller diffusion coefficient of calcium, depending on the number, amount and mobility of the calcium buffers present:

(1) $$D_{app}=D_{{Ca}^{2+}}\frac{\left(1+\frac{D_{mobile}}{D_{{Ca}^{2+}}}\kappa_{mobile}\right)}{\left(1+\kappa_{mobile}+\kappa_{immobile}\right)}\,$$

where, D_Ca^2+^_ is the diffusion coefficient of free calcium in the cytosol, *D*_mobile_ is the diffusion coefficient of mobile buffers, and κ_mobile_ and κ_immobile_ are the calcium buffering capacities of mobile and immobile buffers, respectively (Wagner and Keizer, 1994; Zador and Koch, 1994).

Both mobile and immobile buffers will increase the endogenous κ. By slowing down calcium diffusion, immobile buffers delay the spread of the calcium cloud, prolong the local availability of calcium ions, and decrease the apparent calcium diffusion coefficient. The two immobile calcium buffer backgrounds considered here, κ_immobile_ 15 or 150, both hinder calcium diffusion and reduce *D*_app_ from 220 to 13.8 μm^2^/s (“low immobile buffer background”) and 1.5 μm^2^/s (“high immobile buffer background”), respectively (Equation 1).

Mobile buffers can either increase or decrease the *D*_app_. Consider a system with only immobile buffer and a *D*_app_ of 13.8 μm^2^/s. Now we add a mobile buffer with a diffusion coefficient of 5 μm^2^/s. The new *D*_app_ will depend on the buffering capacity of the mobile species (κ_mobile_), but will always be less than the original *D*_app_ of the system, 13.8 μm^2^/s. Even increasing κ_mobile_ to very large values will not alter the slowing effect of this mobile buffer and will decrease *D*_app_ of the resultant system to the low diffusion coefficient of the mobile buffer. Eq. 1 dictates that adding a mobile calcium buffer to a system can accelerate calcium diffusion (increase *D*_app_), only if the mobile buffer’s diffusion coefficient is larger than the *D*_app_ of the system in the absence of the mobile buffer. Further, if the mobile buffer’s diffusion coefficient is smaller than the original *D*_app_, it will slow calcium diffusion in the system. This means that in the presence of a low immobile buffer background even mobile calcium binding proteins with a diffusion coefficient up to ∼14 μm^2^/s will influence calcium diffusion as if they were immobile; conversely, in the presence of a large amount of immobile buffer only poorly mobile buffers with a diffusion coefficient <1.5 μm^2^/s act as immobile buffers. For a scenario of calcium diffusion in a dendrite, the general limit below which a physically mobile buffer will functionally impact the diffusion of calcium in the system in the same manner as an immobile buffer is given by *D*_m_ < D_Ca^2+^_/(1+κ_immobile_). One should note that equating the impact of mobile and immobile buffers on the apparent diffusion coefficient neglects other consequences of adding a buffer, such as the total buffering capacity. For example, addition of a small amount of immobile buffer or a larger amount of poorly mobile buffer may have the same effect on *D*_app_, but each addition will lead to concomitantly small or large increases in the total endogenous buffering capacity.

The second scenario we consider is a calcium signal with no relevant concentration gradient. Relevant calcium concentration gradients are absent if either the compartment of calcium diffusion is so small that diffusional equilibration is very rapidly achieved (<∼5 ms, e.g., in spines) or if calcium entry is spatially homogenous across a larger compartment (global calcium signal). As there are no gradients, diffusion does not play a role and therefore immobile and mobile buffers affect calcium transients similarly, according to their κ values and the predictions of the single compartment model (Helmchen et al., 1997; Neher, 1998).

**FIGURE 1:**
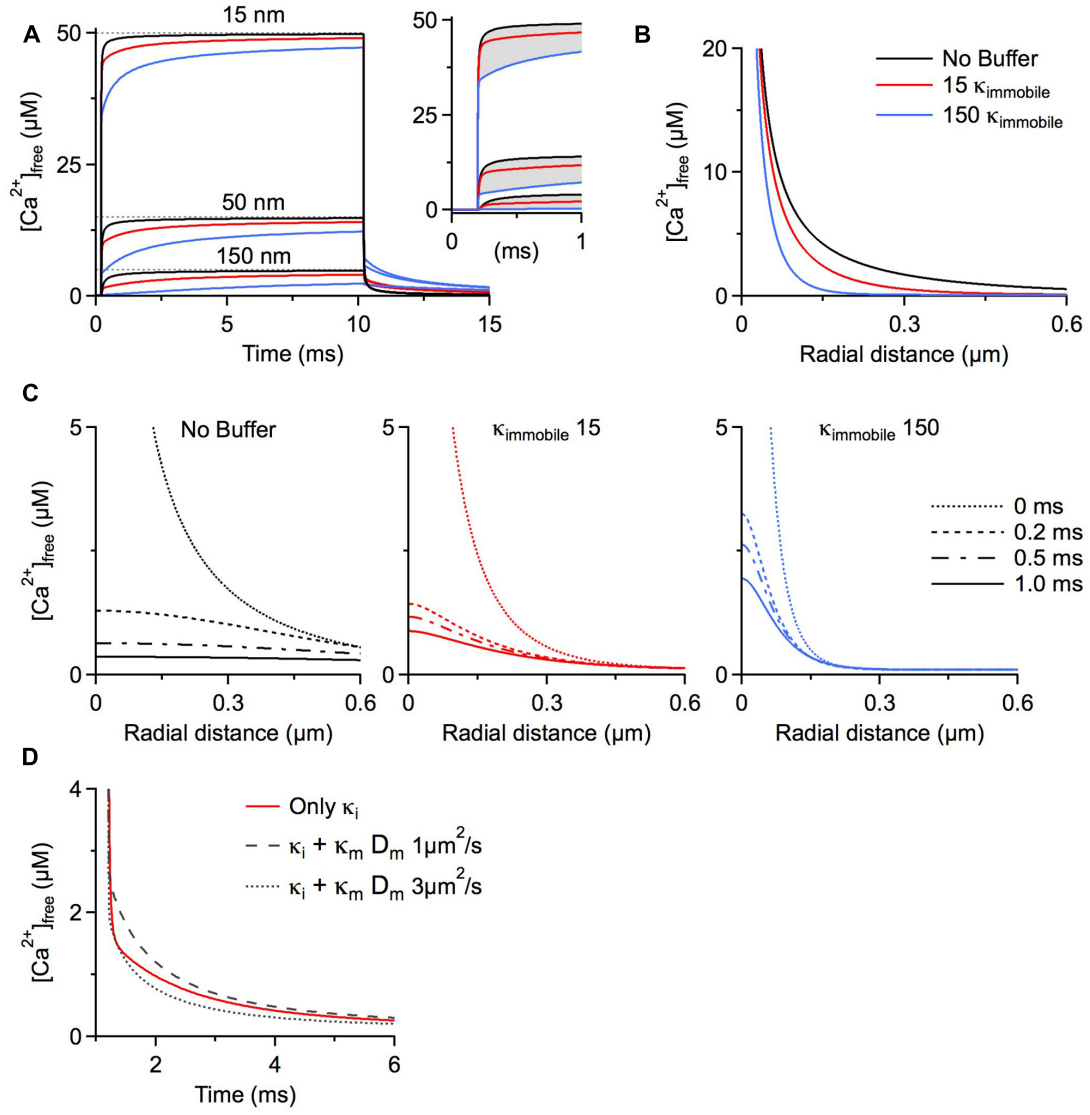
**Simulations were run using CalC (Matveev et al., 2004; http://web.njit.edu/~matveev; Version 6.7.4).** Resting calcium concentration was set at 100 nM. We simulate a calcium current of 0.2 pA through a generic calcium channel. Parameters for the immobile buffer were taken from the experimental results summarized in **Table 1**. **(A)** The presence of immobile buffer slows the arrival at steady state free calcium concentrations at all points in space surrounding a calcium source. The time of channel opening was increased to a non-physiological 10 ms so that the prolonged time course could be better illustrated. However, in the case of high immobile buffering capacity, steady state concentrations were clearly not reached even after 10 ms. In the case of a more physiologically relevant open time of 0.8 ms, the free calcium concentration is even further from reaching a steady state level and the slowing effect of immobile buffers is even more prominent (inset). A similar effect is seen after the channel closes. Calcium is gradually released from immobile buffers, prolonging the return to resting calcium concentrations. **(B)** The localizing effect of immobile buffers on microdomains during calcium entry was plotted for a 1 ms calcium channel opening. At all illustrated points in space, the immobile buffer reduces free calcium concentration; this localizing effect on peak free calcium concentration is known to be similar for a mobile buffer. **(C)** The collapse of a microdomain after the closing of a calcium channel is illustrated for conditions with no buffering (left), a low immobile buffer capacity (center) and a high immobile buffer capacity (right). The spatial calcium gradient is shown immediately before closing (0 ms), and at 0.2, 0.5, and 1.0 ms following channel closure. The strong prolongation of the free calcium domain by the immobile buffer is apparent. With a high immobile buffer capacity, the domain remains above 1 μM free calcium even 1 ms after the channel has closed. This effect is unique to immobile buffers; a mobile buffer will disperse the spatial gradient and speed the local return to resting levels. **(D)** To explore the diffusion limits at which a mobile buffer behaves as a functionally immobile buffer, the prolongation of a calcium microdomain was examined. A calcium source was opened for 1 ms, then the decay of the microdomain at 15 nm from the channel was plotted over time. The initial condition had an immobile buffer with a capacity of 15; under these conditions, the free calcium concentration remains elevated for several milliseconds. When the diffusion coefficient was ≤ 1 μm^2^/s, the lifetime was further increased by the mobile buffer. This indicates that the poorly mobile buffer functions as though it were physically immobile.

Our final scenario examines the function of immobile buffers within microdomains. Calcium microdomains are characterized by a very spatially restricted (<500 nm) standing free calcium concentration gradient rapidly building up around one or several calcium channels after their opening. These gradients persist as long as the channels are open based on a local, steady state equilibrium between calcium binding and calcium and buffer diffusion. Although a steady state is established, there is no chemical equilibrium between the local calcium concentration and mobile buffers because on this small spatial scale diffusion is as rapid as binding kinetics. This is not the case for immobile buffers, which by definition cannot diffuse. Therefore, depletion of calcium-free immobile buffer within the microdomain cannot be replaced by diffusion of calcium-free buffer from remote sites. For this reason immobile buffers do reach chemical equilibrium once the standing gradient of free calcium has fully developed. Further, chemical equilibrium means that at every point in space immobile buffers bind the same number of calcium ions per time as they release and do not affect the standing gradient of free calcium. Simply stated, once calcium microdomains achieve steady state they are not affected by the presence of immobile buffers. But how long does it take to reach steady state? The seminal work by Naraghi and Neher (1997) evaluated the temporal evolution of calcium microdomains. They used millimolar amounts of exogenous calcium chelators, such as EGTA and BAPTA, to probe the spatial extent of microdomain signaling and showed that steady state is reached in only a fraction of a millisecond. They concluded that it is reasonable and accurate to only consider the steady state of a microdomain and neglect immobile buffers. However, as shown below, under physiological conditions, i.e., in the absence of exogenous chelators, it may take 10s of milliseconds to achieve a full steady-state situation even very near the calcium source (**Figure 1A**). In fact, immobile buffers exacerbate the delay to steady state because of the time required before calcium can occupy the local immobile binding sites. Calcium sources such as voltage-gated calcium channels would typically open for less than 1 ms during an action potential (Lee and Elmslie, 1999; Mueller et al., 2005), or <10% of the time it takes to reach steady state, so it can be expected that microdomain steady states are rarely achieved near calcium channels and, accordingly immobile buffers do play a role in shaping calcium microdomains near voltage-gated channels.

When simulating the microdomain with either a low or a high immobile κ, free calcium progresses to steady state substantially more slowly than in the absence of immobile buffering. **Figure 1A** depicts the time course of free calcium around a single calcium channel at three distances, with the dashed line representing the eventual steady state concentration of free calcium. Purely for better illustration of the time course of microdomain calcium we assume a non-physiological single channel open time of 10 ms. Even in the absence of any buffers, the free calcium concentration remains ∼5% below the steady state concentration after 1 ms at distances 50 nm from the channel (physiological open times would be even shorter). Evaluating the free calcium concentration in the presence of immobile buffers at the same time point shows a strong reduction in free calcium concentration because of the slower rise to steady state (**Figure 1A** inset). In fact, immobile buffers reduce the amplitude and restrict the spatial extent of calcium microdomains just as mobile buffers do (**Figures 1A,B**; B shows radial profiles after 1 ms).

After closure of the channel the calcium microdomain rapidly collapses (**Figure 1A**). Mobile buffers accelerate the collapse of the microdomain by shuttling calcium away from the source, thereby strongly reducing the local concentration. This is in contrast to immobile buffers, which not only delay the build-up of the microdomain but also increase microdomain lifetimes after closure of the calcium source because the calcium bound to immobile buffers is locally released, prolonging the return to a resting calcium concentration (Nowycky and Pinter, 1993; see **Figure 1C**, note the localization and prolongation of the microdomain with κ_immobile_ 15 and 150). The outcome of a microdomain on the whole neuron depends on the calcium signaling cascade which is initiated by the microdomain. The question of which calcium signaling cascades might be optimally activated by microdomains with different lifetimes hinges crucially on the on-rates of the different sensor molecules that initiate the cascades, on the different affinities of the sensors, and on the location of the sensor relative to the calcium source. In general, the presence of longer lasting, localized calcium microdomains would allow for the recruitment of calcium signaling cascades initiated by slower acting calcium binding proteins such as parvalbumin. The above discussion of action potential-associated calcium microdomains is also generally applicable to synaptically activated calcium sources. NMDA receptors, for example, are highly permeable to calcium, and are the primary ligand-gated source for postsynaptic calcium. These receptors have longer open times than voltage gated channels, in the range of 2–5 ms (Rosenmund et al., 1995; Lieberman and Mody, 1999). Still, as can be seen in **Figure 1**, adding 150 κ_immobile_ reduces free calcium at 50 nm from the source by ∼25%. Considering that many postsynaptic densities are >100 nm the effect of fixed calcium buffers may be even more pronounced.

Increasing the microdomain lifetime is a function unique to immobile buffers. To define the family of functionally immobile buffers we therefore have to ask under which conditions buffers with a diffusion coefficient >0 will mimic this behavior and prolong the persistence of calcium microdomains in addition to slowing the apparent diffusion coefficient. Poorly mobile buffers increase the lifetime of a microdomain after channel closure by releasing the calcium they had bound during calcium entry while still very close to the channel, due to low buffer mobility. We initially simulated the collapse of a microdomain with a low amount of immobile buffer (κ = 15), and then added a mobile buffer with identical kinetics and affinity, but with a κ_mobile_ of 100. The diffusion coefficient of the mobile buffer was then changed to find a value that would mimic the slow collapse of the microdomain. It turns out that mobile buffers with diffusion coefficients less than 1 μm^2^/s will act as functionally immobile buffers, further prolonging microdomain lifetime (**Figure 1D**). This estimate holds true for a large range of calcium association rates of the mobile buffer. In fact, increasing the on-rate strongly enhances the prolongation effect on the microdomain. However, when the on-rate of a poorly mobile buffer drops below 5 × 10^7^ M^-1^s^-1^, the buffer will cease to mimic the microdomain effect of a physically immobile buffer (in our model). If diffusing freely, only very large mobile calcium binding proteins, >100 kDa, will display diffusion coefficients ≤1 μm^2^/s. Using the higher immobile buffer capacity (κ = 150), the lower limit of the diffusion coefficient that allows a protein to behave as a functionally mobile buffer further drops to 0.2 μm^2^/s (not shown).

It is unlikely that any of the known calcium binding proteins would have such a low diffusion coefficient, so in cells with a high background of immobilized buffer, all known mobile calcium binding proteins would act as both physically and functionally mobile buffers. The degree of microdomain suppression by immobile buffers depends on their concentration, binding kinetics, and channel open time, and therefore cannot be generally predicted. Nevertheless, since the assumed properties of immobile buffers used for these simulations represent probable mean values, our conclusions about the impact of immobile buffers on microdomain signaling at least serve as a good first approximation.

## CONCLUSION

The interplay between mobile and immobile buffers provides neurons with a broad range of options for specifying calcium domains. Immobile buffers play a unique role in shaping calcium signals if relevant concentration gradients are present in dendrites, where they dramatically slow the spread of a calcium cloud. In contrast to common assumptions, immobile buffers are also important regulators of calcium microdomains under physiological conditions when steady state concentration gradients are not expected to occur. Furthermore, depending on the background of immobile buffering capacity, even physically mobile calcium-binding proteins with non-zero diffusion coefficients can function as immobile buffers insofar as they influence the apparent diffusion of calcium and the lifetime of the microdomain. Our simulation is by no means an exhaustive description of the possible parameters that are known to influence free calcium concentration at all spatial and temporal scales. Rather we sought to illustrate a narrower set of circumstances, and in particular the effect of immobile and slowly diffusing buffers on the apparent diffusion of free calcium. The interdependent relationship between the amount of immobile buffer present and the functional behavior of co-expressed, physically mobile buffers highlights the importance of careful experimentation to separate mobile from immobile buffering capacity and highlights the importance of gaining a clearer picture of the kinetics, molecular identity, and prevalence of immobile buffers in neurons.

## Conflict of Interest Statement

The authors declare that the research was conducted in the absence of any commercial or financial relationships that could be construed as a potential conflict of interest.
